# A learning curve in 3D virtual surgical planned orthognathic surgery

**DOI:** 10.1007/s00784-023-05013-2

**Published:** 2023-04-21

**Authors:** Dirk-Melle Beek, Frank Baan, Jeroen Liebregts, Marloes Nienhuijs, Stefaan Bergé, Thomas Maal, Tong Xi

**Affiliations:** 1 Department of Oral and Maxillofacial Surgery, Radboud University Medical Center, Geert Grooteplein 10, 6500 HB Nijmegen, the Netherlands; 2 Radboudumc 3D Lab, Radboud University Medical Center, Geert Grooteplein 10, 6500 HB Nijmegen, the Netherlands

**Keywords:** Learning curve, Orthognathic surgical procedures, Imaging, Three-dimensional, Surgery, Computer-assisted, Maxilla

## Abstract

**Objectives:**

To assess the surgical accuracy of 3D virtual surgical planned orthognathic surgery and the influence of posterior impaction and magnitude of the planned movements on a possible learning curve.

**Materials and methods:**

This prospective cohort study included subjects who underwent bimaxillary surgery between 2016 and 2020 at the Department of Oral and Maxillofacial Surgery of the Radboud University Medical Center, Nijmegen. 3D virtual surgical planning (VSP) was performed with CBCT data and digitalized dentition data. By using voxel-based matching with pre- and postoperative CBCT data the maxillary movements were quantified in six degrees of freedom. The primary outcome variable, surgical accuracy, was defined as the difference between the planned and achieved maxillary movement.

**Results:**

Based on 124 subjects, the surgical accuracy increased annually from 2016 to 2020 in terms of vertical translations (0.82 ± 0.28 mm; *p* = 0.038) and yaw rotations (0.68 ± 0.22°; *p* = 0.028). An increase in surgical accuracy was observed when combining all six degrees of freedom (*p* = 0.021) and specifically between 2016 and 2020 (*p* = 0.004). An unfavorable learning curve was seen with posterior impaction and with a greater magnitude of movements.

**Conclusion:**

The present study demonstrated a significant increase in surgical accuracy annually and therefore supports the presence of a learning curve.

**Clinical relevance:**

Cases with planned maxillary posterior impaction and/or a great magnitude of jaw movements should be transferred from the 3D VSP with extra care to obtain a satisfactory surgical accuracy.

**Supplementary Information:**

The online version contains supplementary material available at 10.1007/s00784-023-05013-2.

## Introduction

Orthognathic surgery is commonly used to correct dentofacial deformities in order to improve oral functions and facial esthetics [1, 2]. Conventionally, surgery was planned with the use of photographs, cephalometry, and plaster models. Nowadays, the planning of orthognathic surgery is based increasingly on a 3-dimensional (3D) virtual approach [3, 4]. This 3D virtual approach requires detailed image data of soft tissue, the bone, and dentition that are obtained by means of stereophotogrammetry, cone beam computed tomography (CBCT) scans, plaster casts, and/or intra-oral scans (IOS). Through image fusion, a 3D virtual augmented head model can be rendered [5].

On a 3D augmented head model, virtual surgical planning (VSP) can be performed. 3D VSP includes the creation of a virtual occlusion, the placement of virtual osteotomy lines, and the virtual repositioning of the maxilla and mandible to an ideal position, based on real-time soft tissue simulation of the subsequent facial profile. The transfer of the 3D VSP to the patient in the operating room is done, in the majority of cases, by using intermediate and final occlusal splints [6]. The splints serve as an interoperative guide for the surgeon to position the maxilla and mandible according to the 3D VSP.

As 3D VSP and operating according to the 3D VSP involve multiple steps and specific insights from the surgeon, they are prone to errors. Previous studies have found that the mean surgical accuracy is < 2 mm for translations and < 4° for rotational movements when comparing the pre- and postoperative position to the planned position of the maxilla and mandible [7–11]. As a surgeon gains more experience in 3D VSP and 3D VSP-based surgery, it is hypothesized that a learning curve may be present regarding the surgical accuracy. A large surgical movement and specific surgical movements such as the (posterior) impaction of the maxilla may also be more difficult to achieve and may be associated with an unfavorable learning curve [12–15]. A recent study established a correlation between time, experience, and surgical accuracy with a landmark-based approach [16]. However, to the best of our knowledge, no studies have investigated the influence of posterior maxillary impaction, the magnitude of the jaw movements, and the type of rotational movements on a possible learning curve. These factors are surgical factors that affect surgical accuracy [12, 17, 18]. Therefore, the present study aimed to assess the surgical accuracy of 3D virtual surgical planned orthognathic surgery and the influence of posterior impaction and magnitude of the planned jaw movements on a possible learning curve with a 3D semi-automated approach. The hypothesis is that there is a difference in surgical accuracy of 3D virtual surgical planned orthognathic surgery throughout the years and that there is an influence of posterior impaction and magnitude of the jaw movements on the learning curve.

## Material and methods

This prospective cohort study included subjects who underwent a bimaxillary osteotomy between 2016 and 2020 at the Department of Oral and Maxillofacial Surgery in Radboud University Medical Centre (Nijmegen, the Netherlands). Subjects were enrolled consecutively. The inclusion criteria were subjects who had a minimum age of 16 and were diagnosed with a non-syndromic dysgnathia that required a bimaxillary osteotomy that consisted of a BSSO and a one-piece Le Fort I osteotomy. Preoperative orthodontic treatment, a minimum of 24 teeth, and the use of 3D VSP were also mandatory. The exclusion criteria were previous orthognathic surgery except for a surgically assisted rapid maxillary expansion (SARME), suboptimal condyle seating on the pre- and/or postoperative CBCT scan, and a history of facial trauma.

This study was performed following the protocol of the World Medical Association Declaration of Helsinki on medical research ethics. This study was approved by the Institutional Review Board (CMO Arnhem-Nijmegen, #2020-6883). All data were pseudonymized prior to analysis.

### Data collection

The CBCT scans were acquired using a standard CBCT scanning protocol (KaVo 3D Exam CBCT scanner (KaVo, Biberach, Germany) with an extended height protocol (FOV 23 × 17 cm at 120 kV and 0.4 mm isotropic voxel size). Subjects were scanned 4 weeks prior to surgery and within 10 days postoperatively. Subjects were scanned while seated in a natural head position with relaxed facial muscles and eyes open. Preoperatively, passive wires were placed in the upper and lower arch to ensure a stable tooth position until surgery. Postoperatively, subjects were scanned in centric occlusion with the surgical splint still in place. The CBCT data were exported in DICOM format.

### 3D planning and surgical procedure

The 3D planning and preoperative workup were performed by a single experienced surgeon. The 3D engineer in the team helped the surgeon to render the 3D augmented virtual head model. In all subjects, IPS CaseDesigner (KLS Martin, Tuttlingen, Germany) was used for 3D VSP. Image data of the soft tissue and bone tissue were imported in IPS CaseDesigner from CBCT (DICOM file). Detailed dentition data were imported as STL files, of either digitalized plaster casts or IOS (TRIOS® 3, 3Shape™, Copenhagen, Denmark).

A 3D augmented head model was created by fusing the CBCT and detailed dentition data. A virtual bimaxillary osteotomy was performed on the head model. Subsequently, the final occlusion was set with the aid of the virtual occlusion tool in IPS CaseDesigner.

The mandible and maxilla were moved to the desired positions to create an esthetically harmonious soft tissue facial profile, as simulated in real-time by the IPS CaseDesigner software. Based on the 3D VSP, an intermediate and final interocclusal wafer were designed and printed to transfer the 3D VSP to the patient in the operating room.

All surgeries were performed by an experienced surgeon or under his direct supervision (TX). The procedure started with nasotracheal intubation. The maxilla was operated first in all cases. The intermediate interocclusal wafer was used to position the maxilla in the desired position. To achieve an accurate vertical control of jaw movements, the distance between a nasion pin and the upper incisor point was measured intra-operatively. The mandible was autorotated until the 3D planned distance between the nasion pin and mesial incisal edge of the right upper central incisor was reached prior to fixation. Subsequently, sagittal split osteotomies were performed according to the Hunsuck modification [19]. The final interocclusal wafer was placed to bring the mandible in the planned position. Miniplates and monocortical screws were used for fixation (KLS 1.5 and 2.0 CMF miniplates and screws, KLS Martin, Tuttlingen, Germany). The interocclusal splint and tight elastics to stabilize the jaws were removed during the first outpatient follow-up 1 week after surgery. After the first week, guiding elastics were used, and postoperative orthodontic treatment was proceeded.

### Analysis of study outcomes

The primary outcome variable was the surgical accuracy in six degrees of freedom: sagittal, vertical, and transverse translations in combination with pitch, roll, and yaw rotations. The surgical accuracy was defined as the 3D spatial difference of the maxilla between the 3D VSP and postoperative CBCT. The primary predictor variable was the year in which the surgery was planned and performed. The secondary predictor variable was the planned posterior impaction of the maxilla. Planned posterior impaction was defined as any cranial movement of both first maxillary molars in the 3D VSP. Covariates such as gender, age, image modality, class of malocclusion, and the magnitude of the planned movements were included. 3D analysis of the surgical accuracy was performed using the OrthoGnathicAnalyser (OGA 2.0) according to the following steps [20]:

1. The upper incisor point, defined as the most mesial point on the incisal edge of the right upper central incisor (11), was indicated and served as a reference point for calculating the translations and rotations.
2. The preoperative 3D virtual head model was orientated in the natural head position that was previously used in the planning software.
3. The pre- and postoperative 3D virtual head models were aligned via voxel-based matching upon the anterior cranial base.
4. The .STL files from the planning were used to translate the preoperative virtually osteotomized maxilla to the 3D planned position in IPS CaseDesigner using surface-based matching. The resulting transformation matrix, containing the translations and rotations, was saved.
5. The maxilla from the pre- and postoperative CBCT data was translated from the preoperative position to the postoperative position using voxel-based matching. Then again, the resulting transformation matrix was saved.
6. The final step was the calculation of the differences between the preoperative planned position and the achieved postoperative position. Therefore, the transformation matrices were converted in the six degrees of freedom.

All analyses were performed twice by the same observer to determine the intra-observer reliability of the measurements.

### Statistics

Statistical analysis was performed in SPSS (version 27, IBM Corp., Armonk, NY, USA). Absolute mean errors and intra-class correlations (ICC) were calculated to assess the measurement errors. Kolmogorov-Smirnov test was used to test the normal distribution of the primary outcome variable. ANOVA with posthoc Bonferroni correction was performed to test for intergroup differences between the consecutive years the surgeries were planned and performed. A vertical bar plot was made to visualize the intergroup differences of the primary outcome variable concerning the consecutive years. A line chart was plotted to visualize the difference in the learning curve between subjects with and without planned posterior impaction. Finally, a linear mixed model analysis was performed to test for the influence of predictors and covariates on the main effect.

## Results

The study population consists of 124 subjects with the following characteristics: 49 male (39.5%), 75 female (60.5%), and a mean age of 29.4 ± 10.7 years old (range 16–58). A total of 13 subjects were diagnosed with class I occlusion (10.5%), 89 with class II malocclusion (71.8%), and 22 with class III malocclusion (17.7%). A significant intergroup difference between the consecutive years in terms of dental imaging modality (*p* < 0.001) and planned roll (*p* = 0.040) was found, as is presented in Table 1 and 2. No other intergroup differences were found.

**Table 1:** Differences in patient demographics between the different years

	2016	2017	2018	2019	2020	*P*-value
n	11	16	39	32	26	
Age Mean ± SD (Range)	31.73±8.44 (20–46)	31.13±11.44 (20–58)	29.38±9.88 (28–52)	30.00±11.41 (17–57)	26.77±11.58 (16–58)	0.631
Gender (%)						
Male (39.5)	7 (63.6)	7 (58.3)	12 (30.8)	9 (28.1)	14 (53.8)	0.091
Female (60.5)	4 (36.4)	9 (41.7)	27 (69.2)	23 (71.9)	12 (46.2)	
Dental imaging modality						
IOS (37.1%)	0 (0)	0 (0)	0 (0)	20 (62.5)	26 (100)	<0.001*
Digitalized plaster casts (62.9%)	11 (100)	16 (100)	39 (100)	12 (37.5)	0 (0)	
Malocclusion class (%)						
Class I (10.5)	2 (18.1)	1 (6.3)	2 (5.1)	4 (12.5)	4 (15.4)	0.520
Class II (71.8)	8 (72.8)	10 (62.5)	28 (71.8)	25 (78.1)	18 (69.2)	
Class III (17.7)	1 (9.1)	5 (31.2)	9 (23.1)	3 (9.4)	4 (15.4)	
Planned posterior impaction						
+ (41.9%)	7 (63.6)	11 (68.8)	21 (53.9)	18 (56.3)	15 (57.7)	0.875
- (58.9%)	4 (36.4)	5 (31.2)	18 (46.1)	14 (43.7)	11 (42.3)	

*P-value < 0.05

**Table 2:** Planned translations and rotations of the maxilla for each year

	Year (mean ± SD)					*P*-value
	2016 (*n* = 11)	2017 (*n* = 16)	2018 (*n* = 39)	2019 (*n* = 32)	2020 (*n* = 26)	
Translations (mm)						
Right-left	0.31±1.50	0.21±0.94	0.53±1.36	0.62±0.84	0.90±1.40	0.428
Anterior-posterior	−3.91±1.44	−3.43±2.04	−3.40±1.78	3.34±1.29	2.94±1.82	0.587
Cranial-caudal	1.62±3.36	0.91±2.49	0.16±2.98	0.42±2.91	−0.41±2.63	0.316
Rotations (°)						
Roll	0.25±2.25	0.34±1.46	0.32±1.23^a^	0.14±1.23	−0.87±2.27^a^	0.040*
Pitch	1.35±3.79	0.92±2.96	1.21±3.12	1.53±3.87	2.35±3.17	0.662
Yaw	0.23±2.25	−0.24±1.50	0.20±1.56	0.44±1.62	0.50±1.36	0.623

*P-value < 0.05

^a^Significant intergroup difference

The mean measurement error ± standard deviation (SD) and ICC results are displayed in Supplementary Material Table S1. The maximum measurement error (0.03 ± 0.96°) was observed for the pitch of the maxilla. The lowest ICC compared to the other degrees of freedom was 0.944 and concerned the roll of the maxilla (Supplementary Material, Table S1), indicating a low measurement error.

The surgical accuracy (mean ± SD) of the maxillary translations and rotations per year is presented in Table 3. Overall, an improvement in surgical accuracy with time was seen in all translations and pitch and yaw rotations. However, only vertical translations between 2016 and 2018 (0.80 ± 0.26 mm; *p* = 0.028) and 2016 and 2020 (0.82 ± 0.28 mm; *p* = 0.038) differed significantly. Besides, the improvement in surgical accuracy was significant for the yaw between 2016 and 2019 (0.64 ± 0.22°; *p* = 0.035) and 2016 and 2020 (0.68 ± 0.22°; *p* = 0.028).

**Table 3:** Surgical accuracy of the translations and rotations of the maxilla for each year

	Year (mean ± SD)					*P*-value
	2016 (*n* = 11)	2017 (*n* = 16)	2018 (*n* = 39)	2019 (*n* = 32)	2020 (*n* = 26)	
Translations (mm)						
Right-left	1.10±0.82	0.87±0.57	0.76±0.74	0.58±0.50	0.71±0.44	0.164
Anterior-posterior	1.30±1.40	1.28±0.79	1.23±0.92	1.16±0.85	1.04±0.79	0.889
Cranial-caudal	1.48±0.76^a,b^	0.94±0.67	0.68±0.66^a^	0.90±1.08	0.67±0.46^b^	0.030*
Rotations (°)						
Roll	0.34±0.22	0.71±0.53	0.84±0.83	0.62±0.49	0.65±0.42	0.169
Pitch	1.56±1.09	1.81±1.40	1.81±1.56	1.46±1.39	1.35±0.85	0.628
Yaw	1.30±0.60^a,b^	0.65±0.46	0.85±0.69	0.66±0.60^a^	0.62±0.61^b^	0.022*

*P-value < 0.05

^a,b^Significant intergroup difference

**Fig. 1:**
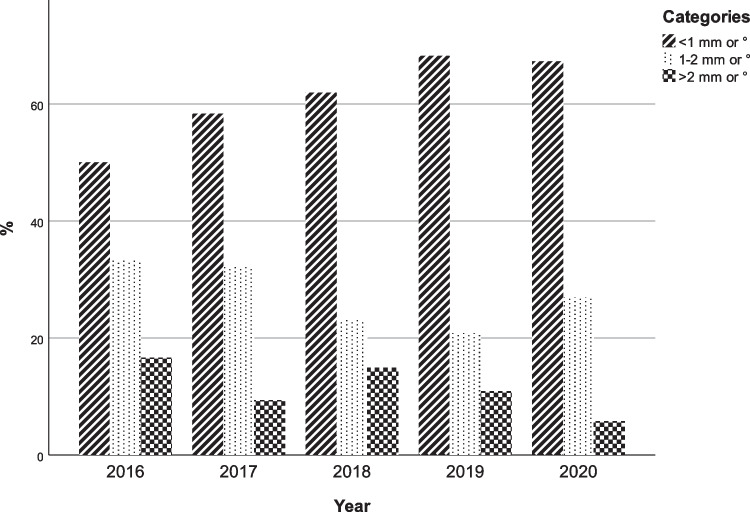
Surgical accuracy of the translations and rotations of the maxilla for each year subdivided in three discrepancy groups. < 1 mm or ° = high accuracy, 1–2 mm or ° = intermediate accuracy and > 2 mm or ° = low accuracy

The surgical accuracy of the maxillary translations and rotations per year was subdivided in three categories: high (< 1 mm or °), intermediate (1–2 mm or °), and low (>2 mm or °) as shown in Table 4 and Fig. 1. In addition, an increase in the number of subjects with a high surgical accuracy and a decrease in the number of subjects with a low surgical accuracy were observed between 2016 and 2020 (*p* = 0.004).

**Table 4:** Surgical accuracy of the translations and rotations of the maxilla for each year subdivided in three discrepancy groups

	High accuracy (< 1 mm or °)	Intermediate accuracy (1–2 mm or °)	Low accuracy (> 2 mm or °)	*P*-value
Number of measurements	470	189	85	
2016 (%)	33 (50)	22 (33.3)	11 (16.7)	0.021*
2017 (%)	56 (58.3)	31 (32.3)	9 (9.4)	
2018 (%)	145 (62.0)	54 (23.1)	35 (14.9)	
2019 (%)	131 (68.2)	40 (20.8)	21 (11.0)	
2020 (%)	105 (67.3)	42 (26.9)	9 (5.8)	

*P-value < 0.05

**Fig. 2:**
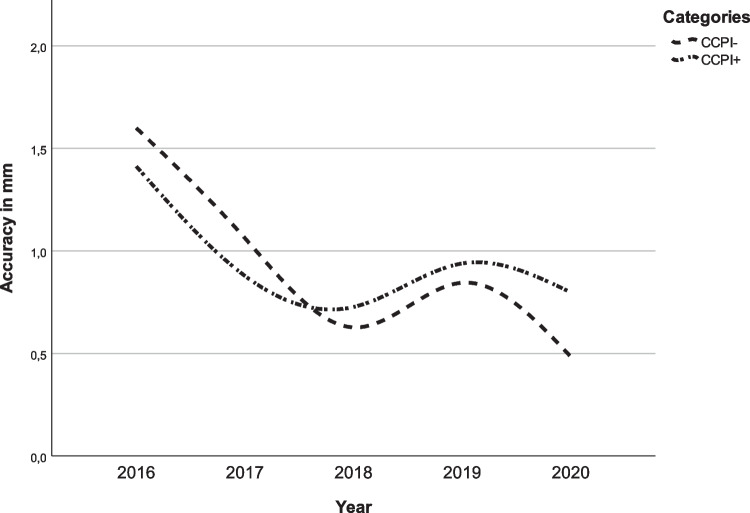
Surgical accuracy of the cranial/caudal movements of the maxilla for each year. CCPI− = cranial-caudal translation in mm without posterior impaction and CCPI+ = cranial-caudal translation in mm with posterior impaction

**Fig. 3:**
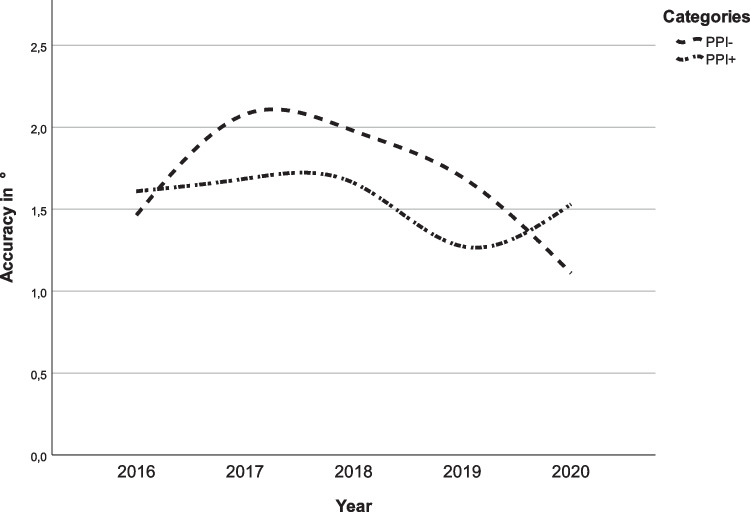
Surgical accuracy of the pitch rotations of the maxilla for each year. PPI− = pitch rotation in ° without posterior impaction and PPI+ = pitch rotation in ° with posterior impaction

Regarding the subgroup with posterior maxillary impaction and without posterior maxillary impaction, a larger improvement in surgical accuracy between 2016 and 2020 was seen in the group without posterior impaction, as is shown in Figs. 2 and 3.

The mixed model analysis demonstrated that the magnitude of planned movements was negatively correlated to the surgical accuracy (*p* < 0.001), and this correlation decreased annually (*p* < 0.001). Next to that, image modality (plaster casts or IOS) had no significant impact on the increase of the surgical accuracy throughout the years (*p* = 0.365).

## Discussion

The present study aimed to assess the surgical accuracy of 3D virtual surgical planned orthognathic surgery and the influence of posterior impaction and magnitude of the planned movements on a possible learning curve with a 3D semi-automated approach. The results of the present study showed a significant increase in surgical accuracy between 2016 and 2020 in terms of vertical translations (0.82 ± 0.28 mm; *p* = 0.038) and yaw rotations (0.68 ± 0.22°; *p* = 0.028). In addition, there was an overall significant increase (*p* = 0.021) in surgical accuracy when subdividing the primary outcome variable in three categories: high (< 1 mm or °), intermediate (1–2 mm or °), and low (> 2 mm or °) and when directly comparing year 2016 with the year 2020 (*p* = 0.004). Besides, a favorable learning curve can be seen in the group that had no planned posterior impaction of the maxilla suggesting that it was easier to gain surgical accuracy in a bimaxillary procedure without posterior impaction. The mixed model analysis also showed that the greater the magnitude of the planned jaw movement, the less accurate the postoperative result was. However, this correlation decreased annually demonstrating the influence of the magnitude of the planned jaw movements on the learning curve.

The present study showed both similar and inconsistent results compared to the study of Antonini et al. [16]. They demonstrated a significant increase in accuracy in a time span of 5 years in horizontal (1.79 ± 0.60 mm to 0.69 ± 0.65 mm, *p* < 0.001), transverse (2.07 ± 1.32 mm to 1.05 ± 0.92 mm, *p* < 0.001), and vertical translations (1.53 ± 0.89 mm to 0.58 ± 0.94 mm, *p* = 0.005). Rotations were excluded in the study. The present study only found a significant increase in vertical translations when comparing the planned and postoperative position of the maxilla (0.82 ± 0.28 mm; *p* = 0.038). However, these contradicting results may be attributed to the differences between both studies in terms of the setting, i.e., different 3D VSP software (Dolphin vs IPS CaseDesigner) and method of analyzing the surgical accuracy (landmark-based vs 3D voxel-based registration). The authors of the present study created the same subdivision of the surgical accuracy in three categories as Antonini et al. to obtain comparable results. A similar trend could be observed as there was a significantly smaller percentage of the low accuracy (> 2 mm) subdivision and a significantly larger percentage of high accuracy (< 1 mm) subdivision present in the more recent years.

Previous studies on learning curves in other surgical fields have shown that reaching proficiency may require several hundred cases [21–24]. However, there is a large variation between surgical procedures, with some studies reporting proficiency after only ten cases [25]. Multiple factors are associated with the learning curve, particularly the previous clinical experience of the surgeon and the surgical difficulty of the procedure. Regardless of the surgical procedure, an improvement or a learning curve is expected. In the present study, a similar trend in the improvement of surgical accuracy was observed. Despite a case load of more than 100 procedures and a period of 5 years, the learning curve in obtaining a higher surgical accuracy has not reached a plateau. A future study to investigate the presence of a learning curve between 5 and 10 years after the implementation of 3D VSP in orthognathic surgery should be envisaged.

The surgical accuracy of the present study was higher at baseline when compared to the study of Antonini et al. This could be attributed to the extensive experience that the surgeon in the present study had in both planning and performing orthognathic surgeries. The surgeon has a double qualification and had completed a 4-year residency program in oral and maxillofacial surgery at the Radboud University Medical Center. At the time of first inclusion, the surgeon had 2 years of experience as the lead consultant in orthognathic surgery and had a case volume of 350 patients. In these 2 years, the surgeon had worked with Maxilim (the forerunner of IPS) software (Medicim, Mechelen, Belgium) for the 3D VSP of orthognathic cases. Nevertheless, a learning curve could still be observed in the present study with the introduction of a new software for 3D VSP. This learning curve could possibly be attributed to the surgeon’s increasing clinical experience and technological advancements in IPS CaseDesigner compared to Maxilim. From this point of view, it could be suggested that the surgical accuracy of an experienced surgeon could be further increased by the introduction of a more intuitive and more automated software for VSP. Performing more surgeries over time is beneficial in this process [26]. Training in VSP and use of the OGA in the evaluation of orthognathic surgery may play a role in the learning curve but this effect was not investigated in the present or in previous studies.

The 3D VSP was translated to the subject in the operating theater using interocclusal splints. A limitation of this approach is that only five degrees of freedom, i.e., left/right, anterior/posterior, pitch, roll, and yaw could be translated directly to the subject.

The vertical movement is challenging to transfer as the control of this movement is determined intra-operatively by the change in distance between the incisal point and the nasion pin (external reference point). The change in distance is affected by multiple factors, i.e., autorotation of the mandible, the thickness of the splint, the maxillary bony interferences, condylar seating, and the amount of maxillary advancement. The removal of bony interferences and adequate condylar seating are the factors that are more operator-dependent. With an increasing case load, a higher surgical accuracy in the vertical positioning of the jaw, could be achieved [21–24]. Autorotation of the mandible, the thickness of the splint, and the magnitude of the maxillary advancement are factors that are less influenced by a learning curve as they are more inherent to the surgical procedure, i.e., the use of a nasion pin and occlusal splint for the vertical positioning of the jaw. Another limitation of this study is the inability to control for the surgeon’s experience in assessing the learning curve associated with virtual planning. Due to the limitations of the present study design, the learning curve associated with virtual planning across levels of surgical experience could not be investigated. The presence of a more favorable learning curve in virtual planning among novice surgeon compared to experienced surgeons could be speculated. Virtual planning may reduce the number of intraoperative adjustments and, thus, could compensate for the lack of clinical experience among nice surgeons. On the other hand, intermediate and experienced surgeons may also benefit from virtual planning by increasing efficiency and reducing operative time. Current literature provides little evidence on the correlation between the learning curve in virtual planning and the level of surgical experience. This should be a topic in future studies.

The strengths of the present study were the use of a semi-automated approach to analyze the surgical accuracy which included both translations and rotations as a primary outcome variable, together with the inclusion of posterior impaction and magnitude of the planned jaw movement in the statistical analysis. In addition, there were little significant intergroup differences between the consecutive years, except for the type of dental imaging modality and the planned roll. However, a mixed model analysis was performed and showed no significant impact of either two factors on the surgical accuracy.

In conclusion, this study demonstrated a significant increase in surgical accuracy over time which supported the presence of a learning curve in 3D virtual surgical planned orthognathic surgery. An unfavorable learning curve was associated with posterior maxillary impaction and a larger magnitude of jaw movement. The awareness of a learning curve in 3D virtual surgical planned orthognathic surgery can be beneficial for oral and maxillofacial residents and surgeons. It provides information on the expected level of surgical accuracy when starting to use 3D VSP, the potential for improvement with practice, and the final level of precision that can be achieved. Authors suggest that future research should focus to examine the entire learning curve over a longer period, to determine when the plateau in surgical accuracy can be reached, and to correlate the level of surgical experience with the learning curve in virtual planning.

## Supplementary Information

Below is the link to the electronic supplementary material.

Supplementary file1 (DOCX 13.1 KB)

## Data Availability

Pseudonymized data are available on request.
