# Supplementary material for: A learning curve in 3D virtual surgical planned orthognathic surgery
Source: Clin Oral Investig. 2023 Apr 21;27(7):3907–15. doi: 10.1007/s00784-023-05013-2 (PMC10329591; doi:10.1007/s00784-023-05013-2)
Supplement: Supplementary file 1 — Supplementary file1 (DOCX 13.1 KB) [file 784_2023_5013_MOESM1_ESM.docx]

**Table S1.** Measurement errors in determining the surgical accuracy of maxillary movements, in terms of absolute measurement errors and intra-class correlation.

|  | | Intergroup differences (n=152)  Mean ± SD | ICC |
| --- | --- | --- | --- |
| **Translations (mm)** | X (left/right) | 0.00±0.17 | 0.989 |
|  | Y (anterior/posterior) | -0.02±0.21 | 0.993 |
|  | Z (cranial/caudal) | 0.00±0.46 | 0.990 |
| **Rotations (⁰)** | Pitch (CW/CCW) | -0.03±0.96 | 0.963 |
|  | Roll (CW/CCW) | -0.01±0.54 | 0.944 |
|  | Yaw (CW/CCW) | 0.02±0.20 | 0.992 |
